# Soluble CTLA-4 as a context-dependent immune checkpoint: biology, biomarker potential, and therapeutic implications

**DOI:** 10.3389/fimmu.2026.1939265

**Published:** 2026-09-08

**Authors:** Xue Cai, Lihua Yu, Xiaoli Liu, Tingting Jiang, Junxiang Li, Zhiyun Yang

**Affiliations:** 1 Center of Integrative Medicine, Beijing Ditan Hospital, Capital Medical University, Beijing, China; 2 Dongfang Hospital, Beijing University of Chinese Medicine, Beijing, China

**Keywords:** alternative splicing, liquid biopsy biomarker, regulatory T cells, soluble CTLA-4, soluble immune checkpoint

## Abstract

Cytotoxic T-lymphocyte-associated antigen 4 (CTLA-4) exists not only as a membrane-bound immune checkpoint but also as a naturally secreted soluble isoform (sCTLA-4), generated primarily through exon 3 skipping. This review focuses specifically on splice-derived sCTLA-4 and examines its biological and clinical significance. sCTLA-4 is shaped by immune state, cellular source, and tissue microenvironment. Mechanistically, sCTLA-4 can restrain B7/CD28-dependent immune activation, while recent preclinical evidence suggests a preferential effect on type 1 immune responses. In autoimmune and inflammatory diseases, longitudinal changes in sCTLA-4 often parallel changes in immune activity, although its performance as a disease-specific biomarker remains limited. In cancer, associations with tumor burden, prognosis, and treatment response are more variable and appear to depend strongly on the surrounding immune context. Thus, circulating sCTLA-4 may be better viewed as a state-dependent immunoregulatory signal than as a universal standalone biomarker. Current evidence does not clearly establish whether its elevation is causal, compensatory, or epiphenomenal. Key translational priorities include detection methods that distinguish sCTLA-4 isoforms and cellular sources, standardized longitudinal sampling, biomarker panels that integrate sCTLA-4 with defined immune phenotypes, and therapeutic strategies tailored to specific immune contexts while preserving peripheral tolerance.

## Graphical Abstract

**Figure:**
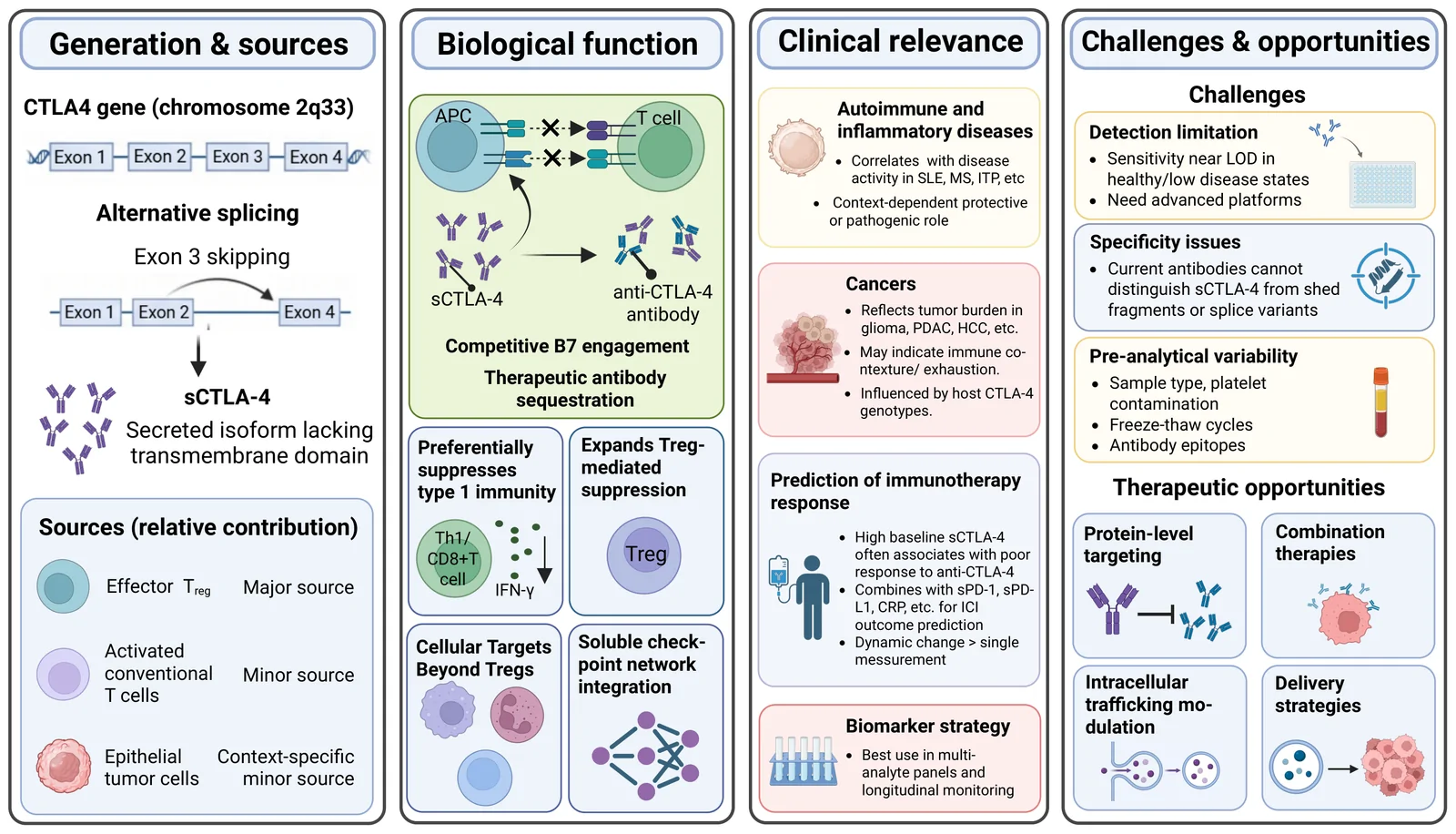

## 1 Introduction

Cytotoxic T-lymphocyte-associated antigen 4 (CTLA-4, CD152) is the prototypic inhibitory immune checkpoint molecule. It is constitutively expressed at high levels on CD4+FoxP3+ regulatory T cells (Tregs) and is transiently upregulated on activated conventional T cells. CTLA-4 outcompetes CD28 for the shared ligands B7-1 (CD80) and B7-2 (CD86) and depletes B7 molecules from antigen-presenting cells through trans-endocytosis (1–3). Therapeutic blockade of CTLA-4 with ipilimumab established immune checkpoint inhibition as a therapeutic class. However, it also revealed a substantial burden of immune-related adverse events (irAEs). These safety concerns have motivated efforts to widen the therapeutic window through local delivery, sustained-release formulations, and rational combination with PD-1/PD-L1 inhibitors (4–8).

Less appreciated, but increasingly central, is a naturally occurring soluble isoform of CTLA-4 (sCTLA-4) generated through alternative splicing that removes the transmembrane-encoding exon 3. As sCTLA-4 contains the B7-binding IgV-like domain without a membrane anchor, it binds extracellular CD80/CD86. This limits CD28 co-stimulatory ligand access and modulates T-cell activation beyond the immunological synapse (9, 10). sCTLA-4 is detectable in the serum of healthy individuals and is markedly dysregulated in autoimmunity, chronic infection, malignancy, and aging. These observations have made it a candidate liquid-biopsy biomarker and a target in its own right (11–15).

Recent advances have renewed interest in the biological and clinical significance of sCTLA-4. A recent mouse genetic and *in vitro* study by Osaki and Sakaguchi reported that sCTLA-4 deficiency preferentially enhanced type 1 immune responses (16). In contrast, impaired function of membrane-bound CTLA-4 (mCTLA-4) produced an autoimmune phenotype biased toward type 2 immunity (16). This immunotype-selective pattern is only backed by a single preclinical study and remains a tentative model instead of a confirmed immunological mechanism. Further animal validation and large-scale human cohort verification are required. sCTLA-4 has also been associated with immune features related to resistance to PD-1/PD-L1 blockade in lung and gastrointestinal cancers (14, 17). In addition, germline CTLA-4 variants are linked to sCTLA-4 expression and clinical outcomes in several malignancies, suggesting a connection between host immunogenetics and disease progression (18, 19). Preclinical studies of engineered sCTLA-4 variants and CTLA-4 trafficking modulators have shown that modifying this axis can alter tumor control and immune-related toxicity (20, 21). Collectively, these findings support sCTLA-4 as a biologically active molecule relevant to immune regulation, longitudinal disease monitoring, and therapeutic targeting.

Based on these advances, we consider that sCTLA-4 is a context-dependent immunoselective regulator with therapeutic potential, rather than a passive serum biomarker. This review summarizes the production mechanisms and biological functions of sCTLA-4. It integrates clinical evidence around relevant biological questions rather than disease types and critically analyzes analytical and therapeutic strategies needed for clinical translation.

## 2 Literature search strategy

This narrative review was informed by a literature search of PubMed, Web of Science Core Collection, and Embase for studies published from 1999 to December 2025. Search terms included combinations of “soluble CTLA-4”, “sCTLA-4”, “CTLA-4 alternative splicing”, “soluble immune checkpoint”, “regulatory T cells”, “autoimmune disease”, “cancer”, “biomarker” and “immunotherapy”. Peer-reviewed original studies directly addressing sCTLA-4 generation, cellular sources, biological functions, clinical associations, detection methods, or therapeutic implications were prioritized. Relevant high-quality reviews were used to provide additional context and identify primary studies. Duplicate publications, conference abstracts without sufficient data, non-peer-reviewed reports, studies lacking direct relevance to sCTLA-4, and articles with insufficient methodological or outcome information were excluded. Reference lists of eligible publications were also screened to identify additional pertinent studies.

## 3 Molecular generation and regulation of sCTLA-4

### 3.1 Gene architecture and alternative splicing

The human CTLA-4 gene comprises four exons encoding, respectively, the signal peptide, the ligand-binding immunoglobulin V-like domain, the transmembrane region, and the cytoplasmic tail. Alternative splicing that skips exon 3 yields a transcript that retains the high-affinity CD80/CD86-binding domain but lacks the membrane anchor. The translated product is secreted as a soluble glycoprotein (9, 10). sCTLA-4 transcripts are detectable in non-stimulated human T cells, indicating that basal alternative splicing contributes to a constitutive low-level soluble pool even in the absence of immune activation (10). A portion of secreted sCTLA-4 is also encapsulated into extracellular vesicles and exosomes. The biological implications of this property, including potential tissue-targeted delivery and an extended functional half-life, have not been fully elucidated to date (9).

Differential exon usage can generate several CTLA-4 isoforms. The main forms are full-length membrane CTLA-4 and soluble CTLA-4, which results from exon 3 skipping. In mice, additional rare isoforms have been reported. These include ligand-independent CTLA-4, which lacks the exon 2–encoded B7-binding domain but retains inhibitory signaling capacity, and a minor isoform containing only exons 1 and 4 (22, 23). However, these isoforms are mainly documented in mice. In humans, only full-length CTLA-4 and sCTLA-4 have been reliably detected at the protein level (9, 24). Because this review focuses on the secreted isoform, the following discussion centers on sCTLA-4.

This species difference is important for translational interpretation. Phenotypes generated by manipulation of the murine CTLA-4 locus may reflect the combined effects of sCTLA-4 and murine-specific isoforms. This is particularly relevant when knockout or editing strategies alter exons or regulatory elements shared by several transcripts. Consequently, changes in T-cell activation, autoimmunity, or tumor immunity cannot automatically be assigned to sCTLA-4 alone. At the same time, the canonical exon 3-skipped sCTLA-4 isoform is conserved in mice and humans, so mouse models remain mechanistically informative when the intervention is demonstrably isoform-specific. Their translational relevance is therefore conditional rather than absolute. Findings should be strengthened by transcript/protein-level confirmation of the affected isoform and validation in human cells or clinical cohorts (16, 22–24).

### 3.2 Transcriptional, epigenetic, genetic, and post-transcriptional control

sCTLA-4 expression is controlled at multiple regulatory layers. Transcriptionally, CTLA-4 activity is shaped by DNA methylation and histone modifications (25). Pharmacological dampening of T-cell activation, such as calcineurin inhibitor therapy in renal transplantation, reduces both CTLA-4 transcription and circulating sCTLA-4 levels. This indicates that systemic T-cell activation state is a major upstream determinant of the sCTLA-4 pool (26). However, the splice-factor program that determines exon 3 skipping in defined T-cell subsets remains incompletely resolved. SR proteins and hnRNPs have been proposed as candidate mediators through which non-coding CTLA-4 variation could influence splicing. However, direct evidence for an sCTLA-4 splicing program specific to effector Tregs or dependent on FOXP3 is currently lacking (18, 19).

Germline variation in CTLA-4 constitutes a distinct, clinically relevant regulatory layer. The CT60 and Jo31 variants, originally characterized in autoimmunity, alter sCTLA-4 serum concentrations and disease susceptibility across multiple ethnic populations (27–29). More recently, rs231726, rs11571317, and rs13384548 have been associated with disease risk, tumor burden, and stepwise sCTLA-4 elevation in pancreatic ductal adenocarcinoma (PDAC) and HCV-related hepatocellular carcinoma (HCC) (18, 19). The mechanistic interpretation of these associations remains incomplete. Most disease-associated CTLA-4 SNPs lie in non-coding regions and could in principle alter sCTLA-4 production through several non-exclusive routes. One route is altered affinity of cis-acting splicing enhancers or silencers for SR proteins and hnRNPs. Other possibilities include changes in mRNA stability mediated by the 3′ UTR or altered looping between enhancers and promoters in T cells and Tregs. Direct evidence linking specific SNPs to defined splicing outcomes is still limited, and this remains an important mechanistic gap that bridges human genetics and sCTLA-4 biology.

Post-transcriptional control of alternative splicing is itself responsive to the microenvironment. Transforming growth factor-β2 (TGF-β2), enriched in many solid tumors, potently induces sCTLA-4 secretion from T cells and correlates with serum sCTLA-4 in melanoma (30). These findings are consistent with TGF-β2-driven sCTLA-4 induction that may reinforce local immune suppression. However, a true reciprocal feedback loop has not yet been demonstrated. Hypoxia and hypoxia-inducible factor-1α (HIF-1α) signaling have been speculatively linked to a shift in CTLA-4 splicing toward the soluble isoform. However, direct evidence that HIF-1α regulates CTLA-4 pre-mRNA splicing is lacking, so this possibility remains conjectural (31). Inflammatory cytokines and paracrine factors from tumor-associated stromal cells may further modulate sCTLA-4 release, both directly and through extracellular vesicles (32).

### 3.3 Cellular sources and microenvironmental accumulation

Although sCTLA-4 can be produced by multiple cell types, effector Tregs are reported to be a major source under both basal and inflammatory conditions (16, 33). This enrichment is consistent with the constitutively high CTLA-4 expression of FoxP3+ Tregs. It also aligns with evidence that T-cell activation state regulates CTLA-4 transcription and the circulating sCTLA-4 pool (1–3, 26). However, the molecular basis for preferential soluble-isoform production in effector Tregs remains incompletely defined. Exon 3 skipping is subject to post-transcriptional regulation, but a Treg-restricted or FOXP3-dependent splice program has not been demonstrated. Thus, current evidence supports effector Tregs as an enriched source without establishing a unique lineage-specific mechanism of sCTLA-4 generation. Functionally, sCTLA-4 produced by Tregs can extend suppressive activity beyond direct cell contact by acting as a diffusible paracrine signal. Activated conventional CD4+ and CD8+ T cells contribute additional sCTLA-4 in proportion to their activation state and the surrounding cytokine milieu (10, 33).

A more recent and clinically relevant observation is that non-lymphoid cells can also contribute to sCTLA-4. sCTLA-4 expression has been detected in normal, dysplastic, and neoplastic oral and oropharyngeal epithelia (34). This suggests that epithelial tumor cells may seed sCTLA-4 in the tumor microenvironment (TME) independently of T-cell infiltration. Such production could promote immune escape at the earliest pre-infiltrative stages of carcinogenesis.

The accumulation of sCTLA-4 in tissue compartments varies according to disease type and the local immune environment. Serum sCTLA-4 is elevated in glioma and correlates with tumor grade (35). Pre-treatment serum sCTLA-4 independently predicts recurrence after radiofrequency ablation in HCV-related HCC (36). In malignant pleural mesothelioma, pleural-effusion sCTLA-4 varies with clinicopathological features but is not a standalone diagnostic or prognostic marker. TGF-β2 and TGF-β1/3 provide stronger signals in that setting (37). Mesothelioma therefore illustrates a more general principle. sCTLA-4 should be interpreted within tissue-specific soluble-checkpoint networks rather than as a universal, isolated marker.

**Figure 1:**
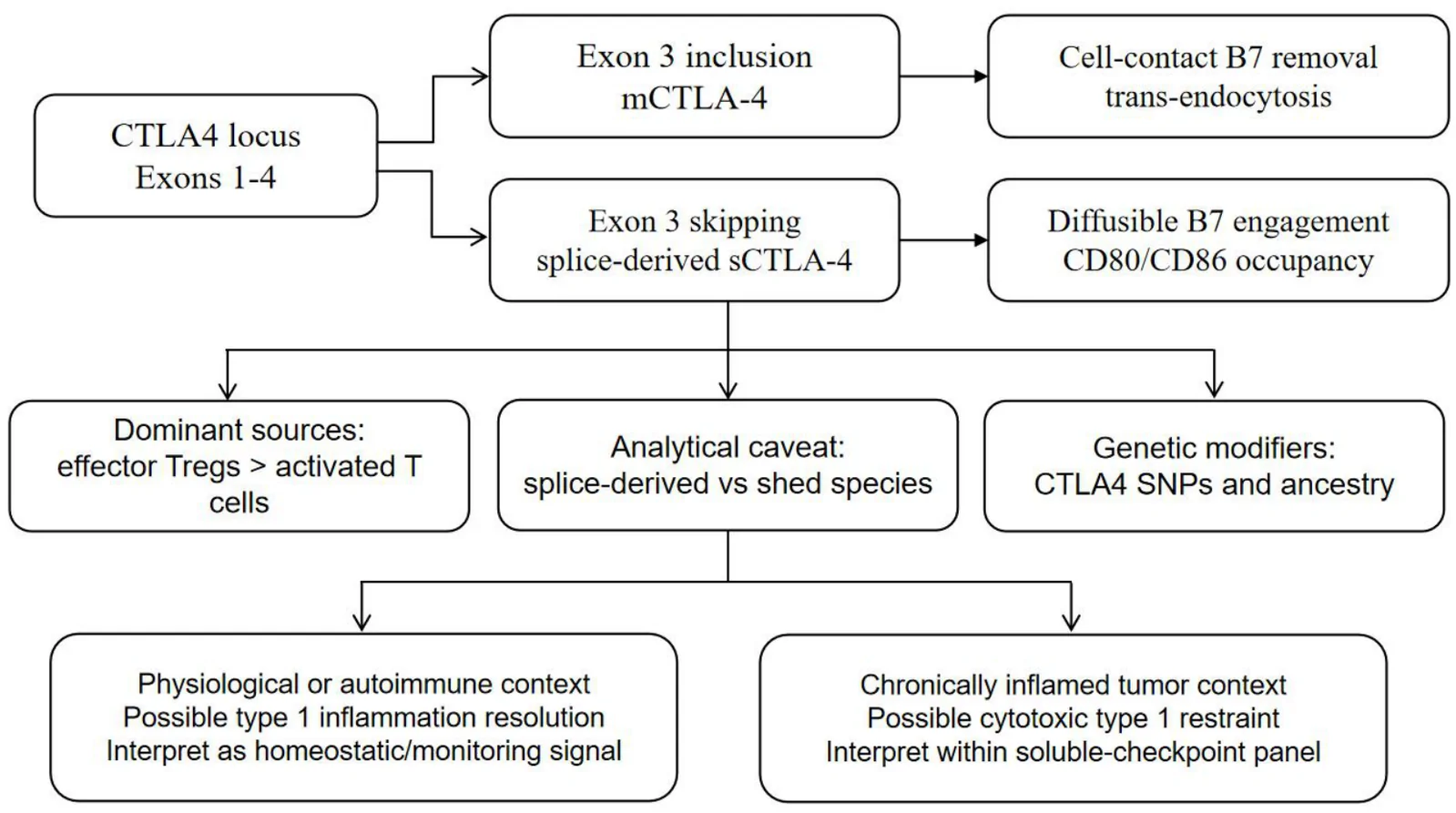
Isoform-resolved model of sCTLA-4 biology and interpretation. Treg, regulatory T cell; mCTLA-4, membrane CTLA-4.

A frequently overlooked analytical question follows directly from this cellular landscape. Most antibody-based platforms quantify sCTLA-4 as a single analyte but use antibodies against the immunoglobulin domain shared by splice-derived and transmembrane isoforms. Because isoform-specific high-affinity reagents are lacking, these platforms do not specifically resolve splice-derived soluble CTLA-4 from the membrane isoform (9). It remains unproven whether membrane CTLA-4 enters the circulation as a proteolytically shed ectodomain, as has been documented for PD-1, PD-L1, and LAG-3. This should therefore be considered a hypothesis instead of a confirmed source of soluble CTLA-4. To the extent that the measured signal is a mixture, conflating these species may attenuate the apparent strength of clinical correlations. These isoform-specific biological and analytical considerations are summarized in **Figure 1**.

## 4 Biological functions of sCTLA-4

For a long time, sCTLA-4 has generally been regarded as a monofunctional molecule that broadly inhibits T cell activation. Recent studies have advanced our understanding of sCTLA-4 biology along four interconnected dimensions. These dimensions include competitive B7 binding and growing evidence for preferential inhibition of type 1 immune responses. They also include regulatory effects on diverse immune cell populations and integration into the wider soluble checkpoint network.

### 4.1 Competitive B7 engagement as the core molecular mechanism

The best-characterized activity of sCTLA-4 is high-affinity engagement of CD80 and CD86, which competitively reduces their availability for CD28-mediated co-stimulation and raises the activation threshold of responding T cells. Unlike mCTLA-4, which operates predominantly at the immunological synapse, sCTLA-4 can act at a distance. It can therefore modulate immune responses in tissue compartments and draining lymph nodes that are spatially separated from the producing cell (33, 38). This ability to sequester B7 ligands outside the immunological synapse provides the mechanistic basis for its effects on T-cell differentiation, myeloid polarization, and soluble-checkpoint interactions discussed below.

Abatacept is a CTLA-4–Fc fusion protein approved for the treatment of rheumatoid arthritis and commonly utilized in experimental research as a soluble CTLA-4 mimic. Its full therapeutic effect stems from B7 sequestration, providing pharmacological proof of concept that competitive B7 engagement alone can mediate immunosuppression (39–42).

### 4.2 Immunotype-selective suppression

One recent preclinical study provided direct evidence that the preferential effect of sCTLA-4 on type 1 immunity depends on its interaction with B7 ligands. Recombinant sCTLA-4 suppressed naïve T-cell proliferation and Th1 differentiation, whereas a Y139A sCTLA-4 mutant with impaired CD80/CD86-binding capacity largely lost this inhibitory activity (16). Moreover, blockade of CD80/CD86 reproduced the suppression of Th1 differentiation, supporting B7 sequestration and consequent attenuation of CD28-dependent priming as a key mechanistic requirement. In contrast, under IL-4-driven polarizing conditions, Th2 differentiation was relatively preserved even at sCTLA-4 concentrations that markedly inhibited Th1 differentiation (16). These findings indicate that the apparent immunotype selectivity does not result from type 1-specific ligand affinity of sCTLA-4. Instead, it likely reflects differences in how type 1 and type 2 differentiation programs depend on B7/CD28 costimulation. Their surrounding cytokine milieus may further shape this selectivity.

Consistent with this model, sCTLA-4-deficient mice exhibited spontaneous expansion and activation of Th1, Th17, Tfh, and Tc1 populations, together with aggravated autoimmunity and defective inflammation resolution (16). By contrast, mice with impaired membrane-bound CTLA-4 but preserved sCTLA-4 showed predominantly type 2-biased immune manifestations (16). Tumor-model studies further support a preferential effect on type 1 effector cells, as tumor-derived sCTLA-4 restrains CD8+ T-cell proliferation, activation, and cytolytic function (38). Although not isoform-specific, dendritic-cell studies also indicate that CTLA-4 engagement can reduce IL-12-dominant antigen-presenting programs (43). It can also favor a more regulatory cytokine environment, which would be expected to disfavor type 1 polarization. Collectively, these observations support a provisional model in which sCTLA-4 preferentially restrains B7/CD28-dependent type 1 immune circuits while relatively sparing type 2 responses. However, because the defining immunotype-selective evidence currently derives predominantly from a single preclinical study, this model requires independent experimental replication and validation in human immune systems.

Building on these preclinical findings, we use an immune phenotype-based framework as a hypothesis-generating tool rather than a validated mechanistic model. Within this framework, the net biological effect of sCTLA-4 may depend on the prevailing immune state and tissue microenvironment. If the preferential restraint of B7/CD28-dependent type 1 responses observed in mice is conserved in humans, increased sCTLA-4 could limit excessive Th1/Tc1-driven inflammation. In selected autoimmune or sterile inflammatory settings, this effect could support immune homeostasis. Conversely, effective tumor control in some settings depends strongly on CD8+/Tc1-mediated type 1 immunity. In such tumors, elevated sCTLA-4 could contribute to immune suppression and may be associated with adverse clinical outcomes. In type 2- or myeloid-dominant environments, however, circulating sCTLA-4 may be less directly related to disease outcome and may instead reflect the broader composition and activation state of the local immune compartment. These interpretations remain hypothetical and require prospective human studies integrating sCTLA-4 measurements with immune-phenotype profiling.

### 4.3 Cellular targets beyond Tregs

#### 4.3.1 Regulatory T cells

Although competitive B7 binding serves as a shared molecular mechanism, this pathway triggers markedly distinct downstream effects across various immune cell subsets. Tregs are an important cellular source of sCTLA-4 and also represent a central functional target (16, 33). sCTLA-4 secreted by Tregs can reinforce the suppressive capacity of Tregs via autocrine/paracrine pathways and block CD80/CD86 signals to prevent destabilization of the Treg phenotype (16, 44).

#### 4.3.2 CD8+ T cells

Beyond Tregs, CD8+ T cells constitute the target with the greatest clinical relevance. Kennedy et al. demonstrated that tumor cell-derived sCTLA-4 suppresses CD8+ T cell proliferation *in vitro*, accelerates tumor growth and experimental metastasis *in vivo*. Characteristically, it restrains intratumoral CD8+ T cells in a non-cytotoxic state instead of directly inducing apoptotic depletion (38). Isoform-specific anti-sCTLA-4 antibody blockade reverses such suppression, enhances the activation and cytolytic capacity of intratumoral CD8+ T cells, and facilitates tumor control (38). Genetic animal studies conducted by Osaki and Sakaguchi further support the selective regulation of Tc1 cells by sCTLA-4, as mice deficient in sCTLA-4 spontaneously accumulate activated Tc1 cells (16). This observation remains preclinical and requires independent validation in human studies.

#### 4.3.3 Dendritic cells, monocytes, and macrophages

Myeloid cells are not merely passive platforms presenting B7 molecules but actively participate in sCTLA-4-related immune regulation. Upon maturation induced by lipopolysaccharide (LPS), Poly(I:C), or inflammatory cytokines, human monocyte-derived dendritic cells (DCs) upregulate both membrane-bound and soluble CTLA-4 (43, 45). In contrast, immature DCs barely express CTLA-4. Engagement of CTLA-4 on DCs shifts cytokine secretion from IL-12 toward IL-10, impairs antigen-presenting capacity, and endows DCs with a partial tolerogenic phenotype (43). Accordingly, DCs are susceptible to paracrine sCTLA-4 and can additionally secrete sCTLA-4 themselves during maturation.

In monocytes and macrophages, tumor-derived sCTLA-4 promotes the accumulation of CD206+ M2- like macrophages within tumors, whereas macrophages from mice lacking sCTLA-4 spontaneously polarize toward an M1 phenotype (16). Tumors with high sCTLA-4 expression remodel myeloid composition, accompanied by decreased numbers of intratumoral F4/80^+^ macrophages and Ly6C^hi^ monocytes (38). These findings extend the regulatory spectrum of sCTLA-4 from lymphoid effector cells to myeloid polarization, indicating that macrophage phenotypic skewing is an underrecognized component of sCTLA-4-driven immune remodeling.

#### 4.3.4 NK and B lymphocytes

Western blot and quantitative real-time PCR have detected both membrane and soluble CTLA-4 isoforms in human NK cell lines. Activated murine and human NK cells upregulate CTLA-4, which suppresses IFN-γ production by NK cells upon stimulation with mature DCs and attenuates NK effector functions (46, 47). Nevertheless, it remains unclear whether sCTLA-4 directly modulates NK cells independent of T cell pathways. NK cell depletion experiments by Kennedy et al. showed that sCTLA-4-mediated suppression of tumor cytotoxicity persisted in the absence of NK cells (38). This suggests that CD8+ T cells, rather than NK cells, are the major effector population restrained by sCTLA-4 in this model.

B lymphocytes also transcribe sCTLA-4 mRNA and express low levels of the corresponding protein (9, 33). sCTLA-4-deficient mice exhibit elevated production of autoantibodies and IgE (16), yet direct evidence supporting an intrinsic B cell regulatory function of sCTLA-4 is still lacking.

#### 4.3.5 Non-immune sources

As described in Section 3.3, epithelial tumor cells (34) and certain non-lymphoid cells under specific conditions contribute to the pool of circulating sCTLA-4. *In vivo* animal studies have directly shown that tumor cell-derived sCTLA-4 regulates CD8+ T-cell function and macrophage polarization (16, 38). These findings confirm that non-Treg-derived sCTLA-4 is biologically active rather than an inert byproduct.

### 4.4 Integration within the soluble checkpoint network

sCTLA-4 does not function in isolation but participates in an interactive soluble checkpoint network, in which the PD-1/PD-L1 axis acts as its primary partner. In colorectal cancer, concurrent elevation of sCTLA-4 and soluble PD-L1 generally predicts markedly poorer prognosis. Mechanistically, sCTLA-4 neutralizes CD80/CD86-mediated co-stimulation, whereas PD-L1 directly suppresses effector T cells by recruiting SHP-2 to deliver inhibitory signals (48, 49). The PD-1/PD-L1 axis also sustains Treg viability via the PI3K–Akt–mTOR and adenosine pathways and upregulates PD-L1 expression on Tregs (44, 50). TGF-β-related signaling mediated by sCTLA-4 can converge with the above regulatory programs responsible for Treg stabilization (51).

## 5 Clinical relevance of sCTLA-4

sCTLA-4 is a multidimensional signal rather than a universal standalone biomarker. It correlates with disease activity, tumor burden, immune-cell composition and activation state, host genetics and treatment response, yet such associations vary markedly across diseases and clinical settings. Therefore, sCTLA-4 should be interpreted within its biological context. **Table 1** summarizes available clinical evidence grouped by core biological questions.

**Table 1:** Summary of sCTLA-4 as a clinical biomarker across diseases, organized by the dominant biological question addressed.

Disease category	Disease	sCTLA-4 direction	Sample/Design	Sample size (n)	Correlation	Clinical utility/question addressed	Ref.
Autoimmune diseases	SLE	↑ Elevated	Serum; cross-sectional	(52) 100 SLE + 40 HC (53) 79 SLE + 40 HC (54) 104 SLE	IFN-α activity; disease activity	Disease activity	(52–54)
Autoimmune diseases	Rheumatoid arthritis	↑/context-dependent	Serum; cross-sectional + intervention	(55) 56 RA + 32 HC (56) 95 RA + 30 OA + 50 HC (paired blood/SF n=37) (57) 36 RA (58) 200 RA + 200 HC (59) 24 RA	Disease activity (variable); MTX response	Disease activity + treatment prediction	(55–59)
Autoimmune diseases	Graves/TED	↑ Elevated	Serum; longitudinal	(29) 93 GO + 93 HC (60) 78 GD/TED + 40 HC; follow-up n=41	TED severity; treatment response	Disease activity	(29, 60)
Autoimmune diseases	Multiple sclerosis	↑ in relapse	Plasma; cross-sectional	(61) 21 MS + 22 NMO + 18 HC	Relapse vs remission	Disease activity	(61)
Autoimmune diseases	Immune cytopenia (paediatric)	↑ active; ↓ remission	Serum; longitudinal	(12) 47 patients + 47 HC	Disease activity	Real-time pharmacodynamic monitoring	(12)
Tumors	Glioma	↑ Elevated	Serum; cross-sectional	(35) 50 glioma + 50 HC	Tumor grade (positive)	Tumor-burden indicator	(35)
Tumors	HCV-related HCC	Stepwise ↑	Serum; cross-sectional	(19) 75 HCC + 75 HCV + 75 HC (36) 88 HCC	High-risk CTLA-4 genotypes; tumor burden	Host genetics + tumor burden	(19, 36)
Tumors	Pancreatic ductal adenocarcinoma	↑ Elevated	Serum; cross-sectional	(18) 125 PDAC + 125 HC	Grade; CA19-9/CEA; rs231726/rs11571317	Host genetics + tumor burden	(18)
Tumors	Colorectal cancer	↑ Elevated	Serum; cross-sectional	(49) 131 CRC	Overall survival (negative); PD-L1 co-elevation	Tumor immune state	(49)
Tumors	Melanoma	↑ Elevated	Serum; pre-treatment	(30) 32 melanoma + 27 HC + 40 SLE (PBMC subset n=12) (62) N/A (perspective) (63) 62 mixed advanced cancers	Anti-CTLA-4 antibody efficacy (negative)	ICI response prediction	(30, 62, 63)
Tumors	NSCLC	Panel-dependent	Plasma; pre/on-treatment	(14) cohorts A/B: 50/149 (tPD-L1 subset n=121); D/E: 42/43	sPD-L1 + sCTLA-4 with PD-1/PD-L1 non-response	ICI response prediction	(14)
Tumors	Gastrointestinal cancer	↑ in resistance	Serum; pre/on-treatment	(17) 36 advanced gastric cancer	sCTLA-4 + CRP; anti-PD-1 resistance	ICI resistance stratification	(17)
Tumors	Bladder cancer	Variable	Serum/multi-omic	(64) TCGA-BLCA n=406; multi-cohort validation	GNG4 co-expression; treatment-modality response	Chemo vs immunotherapy selection	(64)
Tumors	Malignant pleural mesothelioma	Variable	Pleural effusion; cross-sectional	(37) 79 MPM + 34 fibrinous pleuritis	Weaker than TGF-β isoforms	Negative case; panel-dependent	(37)

↑, higher sCTLA-4 concentrations relative to the comparator group (healthy controls, or the inactive/remission state within the same cohort); ↓, lower sCTLA-4 concentrations relative to the comparator group. Sample sizes are reported by cohort where applicable; cohort-specific n values are shown for multi-cohort analyses. Reference 62 is a narrative perspective article without a primary patient cohort and is included for mechanistic interpretation only. SLE, systemic lupus erythematosus; HC, healthy controls; RA, rheumatoid arthritis; OA, osteoarthritis; SF, synovial fluid; MTX, methotrexate; GO, Graves' orbitopathy; GD, Graves' disease; TED, thyroid eye disease; MS, multiple sclerosis; NMO, neuromyelitis optica; HCC, hepatocellular carcinoma; PDAC, pancreatic ductal adenocarcinoma; CRC, colorectal cancer; PBMC, peripheral blood mononuclear cells; NSCLC, non-small-cell lung cancer; tPD-L1, tissue PD-L1; sPD-L1, soluble PD-L1; CRP, C-reactive protein; TCGA-BLCA, The Cancer Genome Atlas bladder urothelial carcinoma cohort; GNG4, G-protein subunit gamma 4; MPM, malignant pleural mesothelioma; ICI, immune checkpoint inhibitor; N/A, not applicable.

### 5.1 sCTLA-4 as an index of disease activity in autoimmune and inflammatory conditions

Across autoimmune and inflammatory disorders, current evidence is more consistent with sCTLA-4 as a state-dependent marker of immune activity than as a disease-specific biomarker. In systemic lupus erythematosus (SLE), elevated serum sCTLA-4 correlates with interferon-α activity and composite disease-activity scores (52–54), supporting an association with the intensity of immune activation. Evidence from longitudinal or within-disease comparisons provides stronger support for dynamic monitoring. Circulating sCTLA-4 decreases in parallel with clinical improvement in Graves’ disease and thyroid eye disease (TED) (29, 60). It is also higher during relapse than remission in multiple sclerosis (61) and falls substantially during remission in paediatric immune cytopenia (12). The convergence of these findings across clinically distinct disorders suggests that sCTLA-4 may reflect a shared immunoregulatory state associated with active disease rather than a pathology-specific signal. However, most available studies are observational and use heterogeneous designs. Therefore, these associations do not establish causality or show that sCTLA-4 provides independent clinical information beyond established measures of disease activity.

Two interpretive challenges complicate this picture. First, the rheumatoid arthritis (RA) literature is genuinely heterogeneous. Some studies report elevated sCTLA-4 in active disease (55, 56), while others find a negative or null association with disease-activity indices (57). This heterogeneity is unlikely to be explained by sampling alone. Plausible contributors include disease duration (early versus established RA), background therapy (untreated versus DMARD-experienced cohorts), and assay differences in epitope specificity. The enrolled disease subset, including seropositive versus seronegative RA, may also contribute. Higher baseline sCTLA-4 has been associated with improved response to methotrexate (57), suggesting that within RA, sCTLA-4 may report a particular immunoregulatory phenotype rather than disease intensity per se.

Second, causality is not established. Elevated sCTLA-4 in active disease has been interpreted as a reactive, IFN-α-driven homeostatic response that dampens overactive T-cell programs (54). Alternatively, it may act as a pathogenic species that competitively displaces endogenous mCTLA-4 from B7 ligands and thereby weakens negative feedback signaling (13, 65). These two interpretations are not mutually exclusive. sCTLA-4 might begin as a compensatory response and become maladaptive under chronic conditions, yet the two models yield opposite predictions about therapeutic targeting. Cross-sectional studies cannot adjudicate between them. Longitudinal trajectories of sCTLA-4 across flares, treatment-induced remissions, and Mendelian-randomization analyses leveraging CTLA-4 polymorphisms are required to clarify direction of effect.

### 5.2 sCTLA-4 in tumors: tumor burden, tumor immune state, and host genetics

#### 5.2.1 sCTLA-4 as a tumor-burden indicator

**Table 1** shows that sCTLA-4 tracks disease extent in some tumors, but this association should not be equated with tumor specificity. Increasing burden may enlarge both tumor-derived and immune-cell-derived sCTLA-4 pools and trigger compensatory immune suppression (16, 33, 34, 38). The circulating level therefore likely integrates tumor mass, immune activation, and negative feedback. Its value is more plausible for longitudinal monitoring alongside established burden markers than for independent staging.

#### 5.2.2 sCTLA-4 as an indicator of the tumor immune state

The variable associations in **Table 1** are consistent with context-dependent biology. Preclinical mouse-model data suggest that, in tumors dependent on type 1 immunity, sCTLA-4 may sequester B7 ligands and restrain CD8+ or Tc1 responses (16, 38). Whether this pattern applies consistently in patients remains uncertain. In Treg- or myeloid-dominant settings, sCTLA-4 may instead mark a broader suppressive network. This possibility helps explain why combinations with other soluble checkpoints or inflammatory mediators generally outperform sCTLA-4 alone. Spatial changes in the CTLA-4/CD28 axis in oral squamous cell carcinoma (66) further indicate that circulating values cannot fully represent tissue-level immune regulation.

#### 5.2.3 sCTLA-4 as a reporter of host immunogenetics

**Table 1** further indicates that host genetics may modify the sCTLA-4 signal. CTLA-4 variants could affect transcription, alternative splicing, or microenvironmental responsiveness (18, 19), so the same concentration may not have identical biological meaning across genetic backgrounds. Because direct variant-to-splicing mechanisms remain unresolved, genotype should currently be used for stratification rather than causal interpretation. Future studies should pair protein measurement with germline analysis and cross-ancestry validation.

#### 5.2.4 The boundaries of sCTLA-4 as a tumor biomarker

Three limits of the current oncology evidence deserve emphasis. Most studies are single-center and cross-sectional, with small absolute differences between groups and substantial overlap of distributions; effect sizes for sCTLA-4 alone are rarely sufficient to drive clinical decision-making. The mesothelioma data (**Table 1**) illustrate that elevations of soluble checkpoints can be biologically real without being diagnostically useful (37). And studies establishing associations between CTLA-4 genotypes and sCTLA-4 levels in specific populations (18, 19) should not yet be generalized across ancestries without independent validation. These limitations do not diminish the conceptual relevance of sCTLA-4; rather, they argue for multi-analyte, multicenter, and longitudinal designs in the next generation of studies.

### 5.3 sCTLA-4 as a predictor of response to immune checkpoint and other therapies

**Figure 2:**
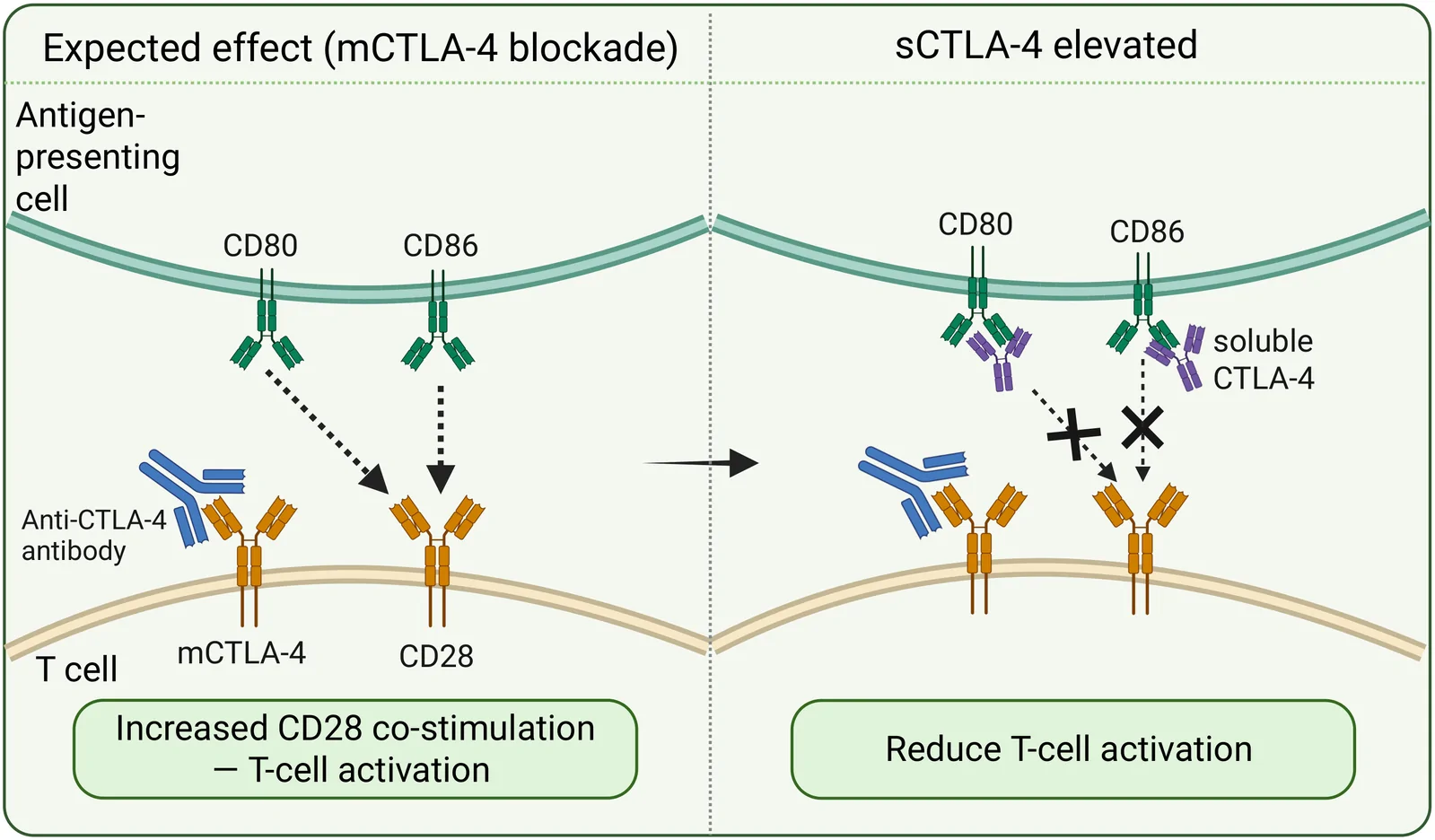
Competitive B7-occupancy model for sCTLA-4-mediated attenuation of anti-CTLA-4 therapy. Under anti-CTLA-4 blockade, CD80/CD86 are expected to become available for CD28-mediated co-stimulation. Elevated sCTLA-4 may continue to occupy CD80/CD86 in the extracellular space, thereby limiting CD28 engagement and weakening T-cell activation.

**Figure 3:**
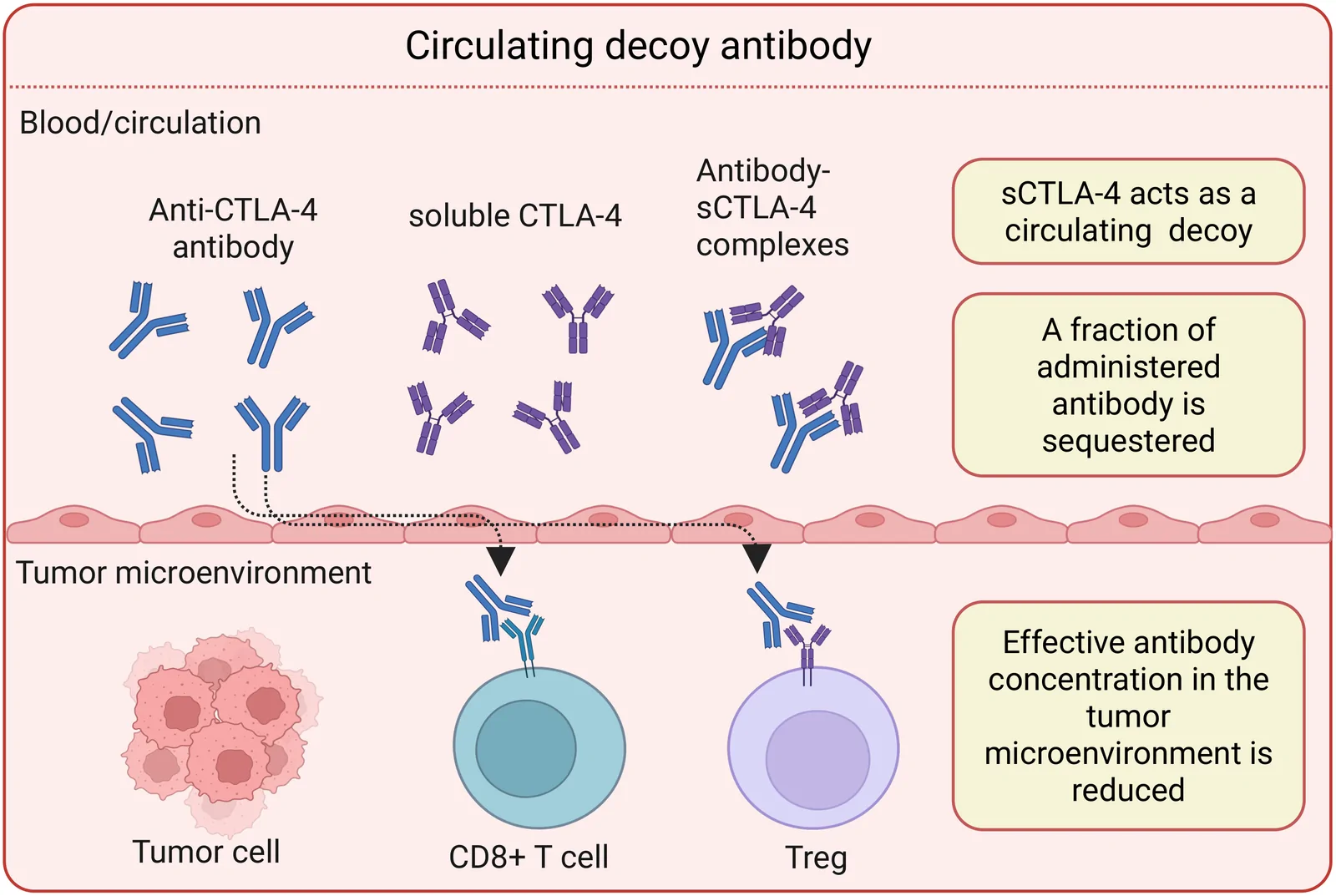
Circulating decoy model for sCTLA-4-mediated reduction of effective anti-CTLA-4 antibody exposure. Circulating sCTLA-4 may bind a fraction of administered anti-CTLA-4 antibodies before they reach the tumor microenvironment. This sequestration could reduce the amount of free antibody available to engage CTLA-4-expressing immune cells and attenuate anti-tumor immune activation.

The most clinically actionable application of sCTLA-4 lies in predicting and monitoring response to therapy. Two non-exclusive mechanisms have been proposed for how baseline sCTLA-4 modifies the activity of anti-CTLA-4 antibodies. On the one hand, sCTLA-4 may compete with mCTLA-4 for CD80/CD86 in the extracellular space. It can occupy B7 ligands that would otherwise become available for CD28 re-engagement after mCTLA-4 blockade. In this model, elevated sCTLA-4 sustains B7 ligand occupancy and dampens net CD28 co-stimulation. On the other hand, sCTLA-4 may absorb a fraction of the administered antibody as a circulating “decoy.” This could reduce the effective antibody concentration available to engage tumor-infiltrating Tregs and effector cells. These mechanisms predict different relationships between sCTLA-4 concentration, antibody pharmacokinetics, and clinical response, and the available human data do not yet distinguish them. Distinguishing the competition and decoy models will require paired measurement of sCTLA-4 concentration and therapeutic-antibody pharmacokinetics across the dosing interval. Measuring free versus sCTLA-4-bound antibody would provide a tractable correlative design for future trials. In melanoma, however, both models predict the empirically observed inverse relationship between baseline sCTLA-4 and clinical response to ipilimumab (30, 62, 67). The corresponding mechanisms are illustrated in **Figures 2** and **3**.

Beyond CTLA-4-directed therapy, sCTLA-4 may also inform PD-1/PD-L1 blockade when measured within a multi-analyte panel that includes other soluble checkpoints or inflammatory markers. Oshima et al. reported that elevated sCTLA-4 combined with high C-reactive protein was associated with anti-PD-1 resistance and outperformed either marker in isolation (17). In NSCLC, the sPD-L1/sCTLA-4 combination helped discriminate responders from non-responders in immune-reactive tumors and complemented tissue PD-L1 immunohistochemistry (14). Across both settings, longitudinal sCTLA-4 kinetics, such as declining concentrations during effective therapy, appear more informative than single-time-point measurements (14, 17). This shift toward dynamic monitoring is methodologically important and is one of the strongest practical arguments for standardizing pre-analytical workflows.

sCTLA-4 may also have predictive or pharmacodynamic value outside ICI therapy, although the evidence remains exploratory. After chemoradiotherapy or radiotherapy for solid tumors, post-treatment elevation of sCTLA-4 has been associated with improved survival, possibly reflecting radiation-induced immunogenic cell death and a compensatory regulatory response (68). In bladder cancer, integration of sCTLA-4 with G-protein subunit gamma 4 (GNG4) expression may stratify patients likely to respond differentially to chemotherapy versus immunotherapy (64). In immune cytopenia, dynamic reductions in sCTLA-4 parallel response to immunosuppressive therapy, supporting its potential use as a real-time pharmacodynamic readout (12).

## 6 Challenges and opportunities in clinical translation

### 6.1 Limitation of detection method

Accurate quantification of sCTLA-4 is a prerequisite for its clinical translation, and the field is constrained by three overlapping analytical limitations. The first is sensitivity. Conventional ELISA platforms approach their lower detection limits at the concentrations typical of healthy individuals and patients in treatment-induced remission (67, 69). Digital ELISA achieves sub-attomolar detection through miniaturized bead-based reactions in femtoliter wells (70). Luminex-based bead arrays enable simultaneous profiling of soluble-checkpoint panels from small liquid-biopsy volumes (60, 71, 72). Surface-enhanced Raman scattering microfluidic biosensors achieve ultra-low detection limits for parallel checkpoint detection, although their clinical translation remains early-stage (71, 73). Importantly, improved sensitivity alone does not ensure greater biomarker validity or comparability across platforms. Assay reports should therefore specify the sample matrix, antibody epitope, calibration range, lower limit of quantification, and whether free or complexed sCTLA-4 is detected.

The second limitation is molecular specificity. Four CTLA-4 splice isoforms exist: full-length membrane-bound CTLA-4, sCTLA-4, liCTLA-4, and the exon 1/4 isoform. All four isoforms are detectable in mice, whereas only full-length CTLA-4 and sCTLA-4 are reliably detected at the protein level in humans. As the sole secreted CTLA-4 isoform, sCTLA-4 possesses only one splice variant generated via exon 3 skipping, which has been identified in humans, mice, rats and dogs (62, 74). Despite there being only one canonical sCTLA-4 splice variant, conventional antibodies cannot distinguish sCTLA-4 from other soluble CTLA-4 fragments in circulation. Consequently, measured signals may not originate purely from splicing-derived sCTLA-4. Therefore, isoform-selective immunoassays, mass spectrometry-based proteomics, and single-molecule sequencing of CTLA-4 pre-mRNA in defined cell populations (75) are required to convert theoretical differentiation of sCTLA-4 species into quantifiable clinical readouts.

This interspecies asymmetry also creates an analytical and model-design limitation. In murine studies, some antibodies, transcript assays, or genetic perturbations do not resolve liCTLA-4 and the exon 1/4 isoform. Such approaches may generate a composite CTLA-4 readout that is not directly comparable with the simpler human isoform repertoire. Preclinical conclusions about sCTLA-4 should therefore specify which CTLA-4 transcripts and proteins are altered or detected and should avoid extrapolating mouse phenotypes to humans without isoform-resolved validation.

The third limitation is pre-analytical. The Hayashi NSCLC study and the gastrointestinal cancer dataset of Oshima et al. show that sCTLA-4 measurements are sensitive to multiple pre-analytical factors (14, 17, 76, 77). These include sample matrix (serum versus plasma), platelet contamination, freeze-thaw history, antibody epitope, and whether the assay detects free or complexed sCTLA-4. These factors are particularly relevant when reported differences between clinical groups are modest, because analytical variation may contribute to apparent differences in effect size across cohorts. They also limit the transferability of numerical cutoffs derived from one study or platform to another. Future studies should therefore prospectively standardize sampling matrix, collection and processing procedures, storage conditions, sampling timepoints, calibrators, and analyte definitions. Without such harmonization, direct cross-study comparisons and meta-analyses will remain difficult, and universal diagnostic or predictive thresholds for sCTLA-4 cannot be reliably established.

Complementary technologies may help place circulating sCTLA-4 measurements into a broader biological context. Flow cytometry can relate circulating sCTLA-4 concentrations to checkpoint expression on defined T-cell populations (67, 78). Radiolabelled immune-checkpoint probes for PET imaging can assess checkpoint distribution non-invasively within tumors (79, 80). These approaches do not replace direct and molecularly specific quantification of sCTLA-4, but they may help clarify its cellular source and relationship to tissue-level immune regulation. Taken together, current analytical limitations suggest that sCTLA-4 measurements are presently more robust for standardized within-study comparisons, longitudinal monitoring, and multi-analyte profiling than for defining universally applicable standalone clinical thresholds.

### 6.2 Therapeutic strategies and translational considerations

sCTLA-4 has dual roles in promoting tumor immune escape and maintaining physiological immune tolerance. This creates a central therapeutic challenge: inhibiting its tumor-promoting activity while preserving its homeostatic functions. Although most emerging regulatory strategies do not directly target sCTLA-4 itself, they indirectly modulate it through other components of the CTLA-4 axis or resistance-related pathways. Their goal is to reduce tumor-associated CTLA-4-mediated suppression while limiting systemic interference with CTLA-4-dependent immune tolerance. **Table 2** summarizes current and emerging therapeutic strategies involving sCTLA-4 or the CTLA-4 axis. These include clinically established CTLA-4-Fc-based immune modulation, protein-level targeting, intracellular trafficking modulation, delivery-based systems, and combination strategies. Most approaches beyond CTLA-4-Fc remain preclinical or in early translational development.

**Table 2:** Therapeutic strategies involving sCTLA-4 or the CTLA-4 axis, grouped by evidence maturity.

Strategy level	Representative approach	Evidence maturity	Key translational issue
Approved immune modulation	Abatacept-like CTLA-4-Fc biology	Clinical use in autoimmunity; indirect relevance to sCTLA-4	May compromise anti-tumor immunity when used during ICI therapy
Protein-level targeting	Anti-sCTLA-4 antibodies; engineered soluble CTLA-4 mutants	Mostly preclinical	Must separate anti-tumor benefit from tolerance disruption
Trafficking modulation	LRBA-CTLA-4 disruption and CTLA-4 degradation	Preclinical/early translational	Effect on soluble versus membrane CTLA-4 requires clarification
Delivery-level targeting	Local delivery; TME-responsive antibody/nanoparticle systems	Preclinical to early clinical rationale	Requires biodistribution, toxicity, and pharmacodynamic endpoints
Combination strategies	Co-targeting CD47, PI3K-gamma, glycolysis, or epigenetic pathways	Conceptual to preclinical	Needs rational biomarker selection and toxicity monitoring

Evidence maturity was added to distinguish established clinical tools from exploratory or preclinical strategies.

At the protein level, conformational epitope-specific anti-sCTLA-4 antibodies selectively neutralize sCTLA-4-mediated immunosuppression in defined antigenic contexts without triggering systemic autoimmunity in preclinical models (13). Engineered soluble CTLA-4 mutants can retain B7-1/B7–2 binding while selectively losing recognition by clinical anti-CTLA-4 antibodies. In preclinical models, these mutants suppressed irAEs while preserving anti-tumor efficacy (20). This offers a structural approach to the abatacept paradox, although clinical validation is still required.

At the intracellular trafficking level, LC427 is an oral small molecule that disrupts the LRBA–CTLA-4 interaction and promotes lysosomal CTLA-4 degradation. In preclinical models, it expands tumor-infiltrating CD8+ T cells and shows anti-tumor activity without inducing colitis-like irAEs (21). Although LC427 does not act directly on sCTLA-4, it offers an alternative route to increase intratumoral CD8+ T-cell activity. It may also be combinable with sCTLA-4-targeted approaches.

At the delivery level, tumor microenvironment-responsive systems, including reactive oxygen species-sensitive bispecific antibody–nanoparticle conjugates, are designed to confine sCTLA-4 blockade to the oxidative TME and limit systemic exposure (81, 82). Local intratumoral or intralymphatic delivery of low-dose anti-CTLA-4 agents has shown that systemic anti-tumor T-cell responses can be elicited without proportionate systemic toxicity (7), but isoform-specific delivery approaches remain early-stage.

Finally, combination-based therapeutic approaches can counter parallel immunosuppressive pathways without directly neutralizing sCTLA-4. Metabolic interventions include PI3Kγ inhibition to reshape macrophage activation (83) and hexokinase 2 inhibition to attenuate lactate-driven immune evasion (84). Epigenetic modifiers such as HDAC inhibitors (25) can also address resistance pathways that act in parallel to sCTLA-4-mediated suppression. These combinations require careful toxicity and pharmacodynamic assessment.

## 7 Conclusions and future perspectives

Soluble CTLA-4 occupies a paradoxical but increasingly intelligible position in immune biology. It is a regulated splice-derived checkpoint molecule with cell-type-specific production, proposed immunotype-selective function, measurable interactions with soluble PD-1/PD-L1 networks, and genetically influenced expression patterns. These features make sCTLA-4 simultaneously attractive and hazardous as a therapeutic target. Indiscriminate inhibition may impair inflammation resolution and peripheral tolerance. By contrast, context-selective modulation could increase intratumoral CD8+/Tc1 proliferation and cytolytic activity while limiting systemic disruption of CTLA-4-dependent tolerance. However, this therapeutic rationale depends on independent experimental replication and confirmation that the proposed immunotype-selective biology is relevant in humans.

Three priorities will determine whether these biomarker and therapeutic applications can be translated clinically. These span two horizons. The first two are near-term, presently achievable priorities in measurement and study design, whereas the third represents a longer-term, still largely hypothesis-driven therapeutic program. First, isoform- and source-resolved detection platforms must replace aggregate sCTLA-4 measurement, so that splice-derived sCTLA-4 and any membrane-derived species, together with individual splice variants, can be interpreted in clinical context. Second, sCTLA-4 must be embedded within multi-analyte, longitudinal liquid-biopsy panels with harmonized pre-analytical workflows. To date, its most clinically informative use has been as part of such panels rather than as a standalone marker. Third, therapeutic development should move from binary checkpoint blockade toward context-selective modulation. Candidate approaches include isoform-selective antibodies, engineered sCTLA-4 mutants, LRBA–CTLA-4 trafficking modulators, and TME-responsive delivery systems. These strategies should be evaluated with pharmacodynamic endpoints that capture sCTLA-4 kinetics, intratumoral B7 availability, Treg dynamics, and type 1/type 2 immune balance. Validation should explicitly span independent animal models and human cohorts.

Conceptualized this way, sCTLA-4 is not a passive serum marker alone but a context-dependent candidate point of intervention within the CTLA-4 axis. Modulating sCTLA-4 selectively may eventually widen the therapeutic window of cancer immunotherapy while preserving homeostatic immune programs that protect patients from treatment-related toxicity. Such modulation should be considered only where isoform, source, immunotype, genotype, and assay context warrant.

## References

[1] Fu J Mao L Jiao Y Mei D Chen Y . Elucidating CTLA-4's role in tumor immunity: a comprehensive overview of targeted antibody therapies and clinical developments. Mol Divers. (2024) 29:5075–84. doi: 10.1007/s11030-024-10917-6 38985379

[2] Halliday N Williams C Kennedy A Waters E Pesenacker AM Soskic B . CD86 is a selective CD28 ligand supporting FoxP3+ regulatory T cell homeostasis in the presence of high levels of CTLA-4. Front Immunol. (2020) 11:600000. doi: 10.3389/fimmu.2020.600000 33363541 PMC7753196

[3] Wing K Onishi Y Prieto-Martin P Yamaguchi T Miyara M Fehervari Z . CTLA-4 control over Foxp3+ regulatory T cell function. Science. (2008) 322:271–5. doi: 10.1126/science.1160062 18845758

[4] Savoia P Astrua C Fava P . Ipilimumab (Anti-Ctla-4 Mab) in the treatment of metastatic melanoma: Effectiveness and toxicity management. Hum Vaccin Immunother. (2016) 12:1092–101. doi: 10.1080/21645515.2015.1129478 26889818 PMC4963052

[5] Harui A Roth MD . Hyaluronidase enhances targeting of hydrogel-encapsulated anti-CTLA-4 to tumor draining lymph nodes and improves anti-tumor efficacy. Gels. (2022) 8. doi: 10.3390/gels8050284 35621582 PMC9141760

[6] Tselikas L de Baere T Isoardo T Susini S Ser-Le Roux K Polrot M . Pickering emulsions with ethiodized oil and nanoparticles for slow release of intratumoral anti-CTLA-4 immune checkpoint antibodies. J Immunother Cancer. (2020) 8. doi: 10.1136/jitc-2020-000579 32571995 PMC7307549

[7] van Pul KM Notohardjo JCL Fransen MF Koster BD Stam AGM Chondronasiou D . Local delivery of low-dose anti-CTLA-4 to the melanoma lymphatic basin leads to systemic T(reg) reduction and effector T cell activation. Sci Immunol. (2022) 7:eabn8097. doi: 10.1126/sciimmunol.abn8097 35857579

[8] Wong SK Beckermann KE Johnson DB Das S . Combining anti-cytotoxic T-lymphocyte antigen 4 (CTLA-4) and -programmed cell death protein 1 (PD-1) agents for cancer immunotherapy. Expert Opin Biol Ther. (2021) 21:1623–34. doi: 10.1080/14712598.2021.1921140 33890832 PMC13093870

[9] Esposito L Hunter KM Clark J Rainbow DB Stevens H Denesha J . Investigation of soluble and transmembrane CTLA-4 isoforms in serum and microvesicles. J Immunol. (2014) 193:889–900. doi: 10.4049/jimmunol.1303389 24928993 PMC4082723

[10] Magistrelli G Jeannin P Herbault N Benoit De Coignac A Gauchat JF Bonnefoy JY . A soluble form of CTLA-4 generated by alternative splicing is expressed by nonstimulated human T cells. Eur J Immunol. (1999) 29:3596–602. doi: 10.1002/(sici)1521-4141(199911)29:11<3596::aid-immu3596>3.0.co;2-y 10556814

[11] Pandey N Mandal M Samanta D Mukherjee G . A nanobody based ultrasensitive electrochemical biosensor for the detection of soluble CTLA-4 - a candidate biomarker for cancer development and progression. Biosens Bioelectron. (2023) 242:115733. doi: 10.1016/j.bios.2023.115733 37820555

[12] Makkeyah SM El-Sherif NH Fathey Hasan M Ibrahim MG Aly NH . Soluble cytotoxic T-lymphocyte antigen-4 (sCTLA-4) in children with immune cytopenia: relation to disease activity. Pediatr Hematol Oncol. (2024) 41:611–9. doi: 10.1080/08880018.2024.2409852 39373368

[13] Dahal LN Basu N Youssef H Khanolkar RC Barker RN Erwig LP . Immunoregulatory soluble CTLA-4 modifies effector T-cell responses in systemic lupus erythematosus. Arthritis Res Ther. (2016) 18:180. doi: 10.1186/s13075-016-1075-1 27487771 PMC4973056

[14] Hayashi H Chamoto K Hatae R Kurosaki T Togashi Y Fukuoka K . Soluble immune checkpoint factors reflect exhaustion of antitumor immunity and response to PD-1 blockade. J Clin Invest. (2024) 134. doi: 10.1172/jci168318 38557498 PMC10977985

[15] Aprilia A Handono K Sujuti H Sabarudin A Winaris N . sCD163, sCD28, sCD80, and sCTLA-4 as soluble marker candidates for detecting immunosenescence. Immun Ageing. (2024) 21:9. doi: 10.1186/s12979-023-00405-0 38243300 PMC10799430

[16] Osaki M Sakaguchi S . Soluble CTLA-4 regulates immune homeostasis and promotes resolution of inflammation by suppressing type 1 but allowing type 2 immunity. Immunity. (2025) 58:889–908.e813. doi: 10.1016/j.immuni.2025.03.004 40168991

[17] Oshima K Shoji H Boku N Hirano H Okita N Takashima A . CRP and soluble CTLA-4 are determinants of anti-PD1 resistance in gastrointestinal cancer. Am J Cancer Res. (2024) 14:1174–89. doi: 10.62347/nqbl9998 38590413 PMC10998742

[18] Hassan M Elhusseny Y Agamy FE Azmy MM Balata M . Soluble CTLA-4 and high-risk genetic variants: a new frontier in pancreatic ductal adenocarcinoma (PDAC) biomarkers. Biochem Biophys Rep. (2025) 44:102304. doi: 10.1016/j.bbrep.2025.102304 41141615 PMC12549928

[19] Hassan M El-Maadawy WH Fahim SA Youssef SM Badran OM Balata M . Integrating CTLA-4 genetics and soluble isoforms for the stratification of HCV-related hepatocellular carcinoma risk and aggressiveness. Int J Mol Sci. (2025) 26. doi: 10.3390/ijms262211067 41303553 PMC12652774

[20] Liu M Wang X Du X Wu W Zhang Y Zhang P . Soluble CTLA-4 mutants ameliorate immune-related adverse events but preserve efficacy of CTLA-4- and PD-1-targeted immunotherapy. Sci Transl Med. (2023) 15:eabm5663. doi: 10.1126/scitranslmed.abm5663 36857433 PMC10501849

[21] Ge X Yu L Zhang L Jia L Meng M He J . Targeting LRBA triggers CTLA-4 degradation and antitumor immunity for cancer immunotherapy. Nat Commun. (2025) 17:623. doi: 10.1038/s41467-025-67365-5 41390689 PMC12815959

[22] Vijayakrishnan L Slavik JM Illés Z Greenwald RJ Rainbow D Greve B . An autoimmune disease-associated CTLA-4 splice variant lacking the B7 binding domain signals negatively in T cells. Immunity. (2004) 20:563–75. doi: 10.1016/s1074-7613(04)00110-4 15142525

[23] Liu SM Sutherland AP Zhang Z Rainbow DB Quintana FJ Paterson AM . Overexpression of the Ctla-4 isoform lacking exons 2 and 3 causes autoimmunity. J Immunol. (2012) 188:155–62. doi: 10.4049/jimmunol.1102042 22124121 PMC3245861

[24] Ueda H Howson JM Esposito L Heward J Snook H Chamberlain G . Association of the T-cell regulatory gene CTLA-4 with susceptibility to autoimmune disease. Nature. (2003) 423:506–11. doi: 10.1038/nature01621 12724780

[25] Dai E Zhu Z Wahed S Qu Z Storkus WJ Guo ZS . Epigenetic modulation of antitumor immunity for improved cancer immunotherapy. Mol Cancer. (2021) 20:171. doi: 10.1186/s12943-021-01464-x 34930302 PMC8691037

[26] Cerci Alkac B Soyoz M Pehlivan M Kilicaslan Ayna T Tatar E Karahan Coven HI . Assessment of CTLA-4 gene expression levels on CD8+ T cells in renal transplant patients and relation with serum sCTLA-4 levels. Biochem Genet. (2025) 63:526–39. doi: 10.1007/s10528-024-10723-7 38467886

[27] Shamala V Asha Devi S . Decoding the genetic influence of CT60 non-coding polymorphism in CTLA-4 gene and sCTLA-4 biomarker with rheumatoid arthritis in the Indian population. Mol Biol Rep. (2024) 51:1023. doi: 10.1007/s11033-024-09949-w 39340674

[28] Huang X Liu X Ye Y Zhang T Mei S Zhu T . Polymorphisms and circulating plasma protein levels of immune checkpoints (CTLA-4 and PD-1) are associated with Posner-Schlossman syndrome in Southern Chinese. Front Immunol. (2021) 12:607966. doi: 10.3389/fimmu.2021.607966 33717091 PMC7943469

[29] Daroszewski J Pawlak E Karabon L Frydecka I Jonkisz A Slowik M . Soluble CTLA-4 receptor an immunological marker of Graves' disease and severity of ophthalmopathy is associated with CTLA-4 Jo31 and CT60 gene polymorphisms. Eur J Endocrinol. (2009) 161:787–93. doi: 10.1530/eje-09-0600 19734241

[30] Khanolkar RC Zhang C Al-Fatyan F Lawson L Depasquale I Meredith FM . TGFβ2 induces the soluble isoform of CTLA-4 - implications for CTLA-4 based checkpoint inhibitor antibodies in Malignant melanoma. Front Immunol. (2021) 12:763877. doi: 10.3389/fimmu.2021.763877 35069536 PMC8767111

[31] Zhang F Guo J Yu S Zheng Y Duan M Zhao L . Cellular senescence and metabolic reprogramming: Unraveling the intricate crosstalk in the immunosuppressive tumor microenvironment. Cancer Commun (Lond). (2024) 44:929–66. doi: 10.1002/cac2.12591 38997794 PMC11492308

[32] Simone R Pesce G Antola P Rumbullaku M Bagnasco M Bizzaro N . The soluble form of CTLA-4 from serum of patients with autoimmune diseases regulates T-cell responses. BioMed Res Int. (2014) 2014:215763. doi: 10.1155/2014/215763 24605322 PMC3925529

[33] Ward FJ Dahal LN Wijesekera SK Abdul-Jawad SK Kaewarpai T Xu H . The soluble isoform of CTLA-4 as a regulator of T-cell responses. Eur J Immunol. (2013) 43:1274–85. doi: 10.1002/eji.201242529 23400950

[34] Clare P Al-Fatyan F Risheh B Nellany K Ward FJ Abu-Eid R . A novel role for the soluble isoform of CTLA-4 in normal, dysplastic and neoplastic oral and oropharyngeal epithelia. Cancers (Basel). (2023) 15. doi: 10.3390/cancers15061696 36980582 PMC10046002

[35] Liu J Tian X Wang Y Kang X Song W . Soluble cytotoxic T-lymphocyte-associated antigen 4 (sCTLA-4) as a potential biomarker for diagnosis and evaluation of the prognosis in glioma. BMC Immunol. (2021) 22:33. doi: 10.1186/s12865-021-00422-y 34006227 PMC8132428

[36] Teng W Jeng WJ Chen WT Lin CC Lin CY Lin SM . Soluble form of CTLA-4 is a good predictor for tumor recurrence after radiofrequency ablation in hepatocellular carcinoma patients. Cancer Med. (2022) 11:3786–95. doi: 10.1002/cam4.4760 35435327 PMC9582685

[37] Okita R Senoo T Mimura-Kimura Y Mimura Y Murakami T Ikeda E . Characterizing soluble immune checkpoint molecules and TGF-β(1,2,3) in pleural effusion of Malignant pleural mesothelioma. Sci Rep. (2024) 14:15947. doi: 10.1038/s41598-024-66189-5 38987362 PMC11236966

[38] Kennedy PT Saulters EL Duckworth AD Lim YJ Woolley JF Slupsky JR . Soluble CTLA-4 attenuates T cell activation and modulates anti-tumor immunity. Mol Ther. (2024) 32:457–68. doi: 10.1016/j.ymthe.2023.11.028 38053333 PMC10861965

[39] Pombo-Suarez M Gomez-Reino JJ . Abatacept for the treatment of rheumatoid arthritis. Expert Rev Clin Immunol. (2019) 15:319–26. doi: 10.1080/1744666x.2019.1579642 30730220

[40] Hervey PS Keam SJ . Abatacept. BioDrugs. (2006) 20:53–61. doi: 10.2165/00063030-200620010-00004 16573350

[41] Rubbert-Roth A Enejosa J Pangan AL Haraoui B Rischmueller M Khan N . Trial of upadacitinib or abatacept in rheumatoid arthritis. N Engl J Med. (2020) 383:1511–21. doi: 10.1056/nejmoa2008250 33053283

[42] Cope AP Jasenecova M Vasconcelos JC Filer A Raza K Qureshi S . Abatacept in individuals at high risk of rheumatoid arthritis (APIPPRA): a randomised, double-blind, multicentre, parallel, placebo-controlled, phase 2b clinical trial. Lancet. (2024) 403:838–49. doi: 10.1016/s0140-6736(23)02649-1 38364839

[43] Laurent S Carrega P Saverino D Piccioli P Camoriano M Morabito A . CTLA-4 is expressed by human monocyte-derived dendritic cells and regulates their functions. Hum Immunol. (2010) 71:934–41. doi: 10.1016/j.humimm.2010.07.007 20650297

[44] Chen JX Yi XJ Gao SX Sun JX . The possible regulatory effect of the PD-1/PD-L1 signaling pathway on Tregs in ovarian cancer. Gen Physiol Biophys. (2020) 39:319–30. doi: 10.4149/gpb_2020011 32902402

[45] Wang XB Fan ZZ Anton D van Vollenhoven A Ni ZH Chen XF . CTLA4 is expressed on mature dendritic cells derived from human monocytes and influences their maturation and antigen presentation. BMC Immunol. (2011) 12:21. doi: 10.1186/1471-2172-12-21 21414236 PMC3070687

[46] Stojanovic A Fiegler N Brunner-Weinzierl M Cerwenka A . CTLA-4 is expressed by activated mouse NK cells and inhibits NK cell IFN-γ production in response to mature dendritic cells. J Immunol. (2014) 192:4184–91. doi: 10.4049/jimmunol.1302091 24688023

[47] Lougaris V Tabellini G Baronio M Patrizi O Gazzurelli L Mitsuiki N . CTLA-4 regulates human natural killer cell effector functions. Clin Immunol. (2018) 194:43–5. doi: 10.1016/j.clim.2018.06.010 29966715

[48] Pulanco MC Madsen AT Tanwar A Corrigan DT Zang X . Recent advancements in the B7/CD28 immune checkpoint families: new biology and clinical therapeutic strategies. Cell Mol Immunol. (2023) 20:694–713. doi: 10.1038/s41423-023-01019-8 37069229 PMC10310771

[49] Omura Y Toiyama Y Okugawa Y Yin C Shigemori T Kusunoki K . Prognostic impacts of tumoral expression and serum levels of PD-L1 and CTLA-4 in colorectal cancer patients. Cancer Immunol Immunother. (2020) 69:2533–46. doi: 10.1007/s00262-020-02645-1 32577816 PMC11027465

[50] Zhang T Zhao J Zheng T Fu W Ma T . Adenosine 2A receptor antagonists promote lymphocyte proliferation in sepsis by inhibiting Treg expression of PD-L1 in spleen. Immunology. (2024) 171:566–82. doi: 10.1111/imm.13744 38158796

[51] Zhulai G Oleinik E . Targeting regulatory T cells in anti-PD-1/PD-L1 cancer immunotherapy. Scand J Immunol. (2022) 95:e13129. doi: 10.1111/sji.13129 34936125

[52] Liu MF Wang CR Chen PC Fung LL . Increased expression of soluble cytotoxic T-lymphocyte-associated antigen-4 molecule in patients with systemic lupus erythematosus. Scand J Immunol. (2003) 57:568–72. doi: 10.1046/j.1365-3083.2003.01232.x 12791095

[53] Wong CK Lit LC Tam LS Li EK Lam CWK . Aberrant production of soluble costimulatory molecules CTLA-4, CD28, CD80 and CD86 in patients with systemic lupus erythematosus. Rheumatol (Oxford). (2005) 44:989–94. doi: 10.1093/rheumatology/keh663 15870153

[54] Dahal LN Barker RN Ward FJ . The soluble isoform of CTLA-4 correlates with interferon-α activity in systemic lupus erythematosus. J Rheumatol. (2020) 47:302–4. doi: 10.3899/jrheum.190678 31787598

[55] Cao J Zou L Luo P Chen P Zhang L . Increased production of circulating soluble co-stimulatory molecules CTLA-4, CD28 and CD80 in patients with rheumatoid arthritis. Int Immunopharmacol. (2012) 14:585–92. doi: 10.1016/j.intimp.2012.08.004 22917707

[56] Wan B Nie H Liu A Feng G He D Xu R . Aberrant regulation of synovial T cell activation by soluble costimulatory molecules in rheumatoid arthritis. J Immunol. (2006) 177:8844–50. doi: 10.4049/jimmunol.177.12.8844 17142787

[57] Malik AE Slauenwhite D McAlpine SM Hanly JG Marshall JS Issekutz TB . Differences in IDO1(+) dendritic cells and soluble CTLA-4 are associated with differential clinical responses to methotrexate treatment in rheumatoid arthritis. Front Immunol. (2024) 15:1352251. doi: 10.3389/fimmu.2024.1352251 38840915 PMC11150726

[58] Torres-Carrillo N Ontiveros-Mercado H Torres-Carrillo NM Parra-Rojas I Rangel-Villalobos H Ramirez-Duenas MG . The -319C/+49G/CT60G haplotype of CTLA-4 gene confers susceptibility to rheumatoid arthritis in Mexican population. Cell Biochem Biophys. (2013) 67:1217–28. doi: 10.1007/s12013-013-9640-6 23703660

[59] Hall C Pleasance J Hickman O Kirkham B Panayi GS Eggleton P . The biologic IRL201805 alters immune tolerance leading to prolonged pharmacodynamics and efficacy in rheumatoid arthritis patients. Int J Mol Sci. (2024) 25. doi: 10.3390/ijms25084394 38673979 PMC11049849

[60] Yin Q Zhu T Song D Fang S Zhou H Guan H . Soluble immune checkpoints associated with disease activity and treatment response in GD and TED. J Clin Endocrinol Metab. (2024) 110:1537–49. doi: 10.1210/clinem/dgae763 39475457

[61] Wang H Wang K Zhong X Dai Y Wu A Li Y . Plasma sCD28, sCTLA-4 levels in neuromyelitis optica and multiple sclerosis during relapse. J Neuroimmunol. (2012) 243:52–5. doi: 10.1016/j.jneuroim.2011.11.010 22177277

[62] Azimnasab-Sorkhabi P Soltani-Asl M Bouhajra M Ansa-Addo EA Kfoury Junior JR . Soluble CTLA-4 - A confounding factor in CTLA-4 based checkpoint immunotherapy in cancer. Immunol Lett. (2024) 272:106965. doi: 10.1016/j.imlet.2024.106965 39667581

[63] Cohen M Shamay Y Czamanski-Cohen J Shulman K Keren Rosenberg S Abu-Amna M . Linkage between psychological factors and response to immune checkpoint inhibitor therapy: a preliminary study. Cells. (2023) 12:2471. doi: 10.3390/cells12202471 37887315 PMC10605722

[64] Duan L Liu X Luo Z Zhang C Wu C Mu W . G-Protein subunit gamma 4 as a potential biomarker for predicting the response of chemotherapy and immunotherapy in bladder cancer. Genes (Basel). (2022) 13. doi: 10.3390/genes13040693 35456499 PMC9027884

[65] Hossen MM Ma Y Yin Z Xia Y Du J Huang JY . Current understanding of CTLA-4: from mechanism to autoimmune diseases. Front Immunol. (2023) 14:1198365. doi: 10.3389/fimmu.2023.1198365 37497212 PMC10367421

[66] Feldmeier F Weber M Pacelli F Vogl C Glajzer J Trumet L . Investigation of the CTLA-4-CD28 axis in oral squamous cell carcinoma. J Clin Med. (2025) 14. doi: 10.3390/jcm14145171 40725864 PMC12294955

[67] Machiraju D Wiecken M Lang N Hulsmeyer I Roth J Schank TE . Soluble immune checkpoints and T-cell subsets in blood as biomarkers for resistance to immunotherapy in melanoma patients. Oncoimmunology. (2021) 10:1926762. doi: 10.1080/2162402x.2021.1926762 34104542 PMC8158029

[68] Liu Q Hu P Deng G Zhang J Liang N Xie J . Soluble cytotoxic T-lymphocyte antigen 4: a favorable predictor in Malignant tumors after therapy. Onco Targets Ther. (2017) 10:2147–54. doi: 10.2147/ott.s128451 28442923 PMC5396933

[69] Xing Y Liu J Sun S Ming T Wang Y Luo J . New electrochemical method for programmed death-ligand 1 detection based on a paper-based microfluidic aptasensor. Bioelectrochemistry. (2021) 140:107789. doi: 10.1016/j.bioelechem.2021.107789 33677221

[70] Kan CW Tobos CI Rissin DM Wiener AD Meyer RE Svancara DM . Digital enzyme-linked immunosorbent assays with sub-attomolar detection limits based on low numbers of capture beads combined with high efficiency bead analysis. Lab Chip. (2020) 20:2122–35. doi: 10.1039/d0lc00267d 32391827

[71] Wuethrich A Rajkumar AR Shanmugasundaram KB Reza KK Dey S Howard CB . Single droplet detection of immune checkpoints on a multiplexed electrohydrodynamic biosensor. Analyst. (2019) 144:6914–21. doi: 10.1039/c9an01450k 31657376

[72] Gelibter A Tuosto L Asquino A Siringo M Sabatini A Zizzari IG . Anti-PD1 therapies induce an early expansion of Ki67(+)CD8(+) T cells in metastatic non-oncogene addicted NSCLC patients. Front Immunol. (2024) 15:1483182. doi: 10.3389/fimmu.2024.1483182 39744631 PMC11688303

[73] Reza KK Sina AA Wuethrich A Grewal YS Howard CB Korbie D . A SERS microfluidic platform for targeting multiple soluble immune checkpoints. Biosens Bioelectron. (2019) 126:178–86. doi: 10.1016/j.bios.2018.10.044 30415152

[74] Tagawa M Yamamoto Y Shimbo G Iguchi A Xuan X Tomihari M . Gene and protein expression of a soluble form of CTLA-4 in a healthy dog. J Vet Med Sci. (2017) 79:871–5. doi: 10.1292/jvms.16-0583 28392507 PMC5447975

[75] Wang J Wong WP Link EO Olivares S Adelman CT Henkel AS . Single well, single-common primer pair, dual probe, duplex qPCR assay for the quantification of mRNA splicing variants. Biol Methods Protoc. (2021) 6:bpab002. doi: 10.1093/biomethods/bpab002 33655078 PMC7903517

[76] Pitts SC Schlom J Donahue RN . Soluble immune checkpoints: implications for cancer prognosis and response to immune checkpoint therapy and conventional therapies. J Exp Clin Cancer Res. (2024) 43:155. doi: 10.1186/s13046-024-03074-z 38822401 PMC11141022

[77] Chen L Chao Y Li W Wu Z Wang Q . Soluble immune checkpoint molecules in cancer risk, outcomes prediction, and therapeutic applications. biomark Res. (2024) 12:95. doi: 10.1186/s40364-024-00647-0 39218939 PMC11368031

[78] Shibru B Fey K Fricke S Blaudszun AR Furst F Weise M . Detection of immune checkpoint receptors - A current challenge in clinical flow cytometry. Front Immunol. (2021) 12:694055. doi: 10.3389/fimmu.2021.694055 34276685 PMC8281132

[79] John C Jain K Masanam HB Narasimhan AK Natarajan A . Recent trends and opportunities for the targeted immuno-nanomaterials for cancer theranostics applications. Micromachines (Basel). (2022) 13. doi: 10.3390/mi13122217 36557516 PMC9781111

[80] Lesniak WG Chatterjee S Gabrielson M Lisok A Wharram B Pomper MG . PD-L1 detection in tumors using [(64)Cu]Atezolizumab with PET. Bioconjug Chem. (2016) 27:2103–10. doi: 10.1021/acs.bioconjchem.6b00348 27458027 PMC5289227

[81] Wang D Wang T Yu H Feng B Zhou L Zhou F . Engineering nanoparticles to locally activate T cells in the tumor microenvironment. Sci Immunol. (2019) 4:eaau6584. doi: 10.1126/sciimmunol.aau6584 31300478

[82] Desai SA Patel VP Bhosle KP Nagare SD Thombare KC . The tumor microenvironment: shaping cancer progression and treatment response. J Chemother. (2025) 37:15–44. doi: 10.1080/1120009x.2023.2300224 38179655

[83] Carnevalli LS Taylor MA King M Coenen-Stass AML Hughes AM Bell S . Macrophage activation status rather than repolarization is associated with enhanced checkpoint activity in combination with PI3Kγ inhibition. Mol Cancer Ther. (2021) 20:1080–91. doi: 10.1158/1535-7163.mct-20-0961 33785652

[84] Guo D Tong Y Jiang X Meng Y Jiang H Du L . Aerobic glycolysis promotes tumor immune evasion by hexokinase2-mediated phosphorylation of IκBα. Cell Metab. (2022) 34:1312–1324.e1316. doi: 10.1016/j.cmet.2022.08.002 36007522

